# Immediate versus Delayed Sequential Bilateral Cataract Surgery: A Systematic Review and Meta-Analysis

**DOI:** 10.1371/journal.pone.0131857

**Published:** 2015-06-29

**Authors:** Monali S. Malvankar-Mehta, Yufeng Nancy Chen, Sangita Patel, Angela Pui-Kei Leung, Man Mohan Merchea, William G. Hodge

**Affiliations:** 1 Department of Ophthalmology, Schulich School of Medicine and Dentistry, University of Western Ontario, London, ON, Canada; 2 Department of Epidemiology and Biostatistics, Schulich School of Medicine and Dentistry, University of Western Ontario, London, ON, Canada; 3 Schulich School of Medicine and Dentistry, University of Western Ontario, London, ON, Canada; 4 Faculty of Health Sciences, University of Western Ontario, London, ON, Canada; Medical University Graz, AUSTRIA

## Abstract

**Background:**

Immediately sequential bilateral cataract surgery (ISBCS), the cataract surgery that is performed in both eyes simultaneously, is gaining popularity worldwide compared to the traditional treatment paradigm: delayed sequential bilateral cataract surgery (DSBCS), the surgery that is performed in each eye on a different day as a completely separate operation. ISBCS provides advantages to patients and patients’ families in the form of fewer hospital visits. Additionally, patients enjoy rapid rehabilitation, lack of anisometropia - potentially reducing accidents and falls, and avoid suboptimal visual function in daily life. The hospital may benefit due to lower cost.

**Objective:**

To perform a systematic review and meta-analysis to evaluate ISBCS and DSBCS.

**Data Sources:**

Databases including MEDLINE, EMBASE, BIOSIS, CINAHL, Health Economic Evaluations Database (HEED), ISI Web of Science (Thomson-Reuters) and the Cochrane Library were searched.

**Participants:**

Not applicable.

**Methods:**

Literature was systematically reviewed using EPPI-Reviewer 4 gateway. Meta-analysis was conducted using STATA v. 13.0. Standardized mean difference (SMD) and 95% confidence intervals (CI) were calculated and heterogeneity was assessed using *I^2^* statistics. Fixed-effect and random-effect models were computed based on heterogeneity. Meta-analysis was done by instrument used to calculate utility score.

**Results:**

In total, 9,133 records were retrieved from multiple databases and an additional 128 records were identified through grey literature search. Eleven articles with 3,657 subjects were included for analysis. Our meta-analysis results indicated significant improvement in post-operative utility score using TTO, EQ5D, HUI3, VF-7, and VF-14 and a non-significant improvement using Catquest questionnaire for both surgeries. For ISBCS versus DSBCS, utility-specific fixed-effect model provided an overall SMD of the utility score using the TTO method as 0.12 (95% CI: -0.15, 0.40), EQ5D as 0.14 (95% CI: -0.14, 0.41), HUI3 as 0.12 (95% CI: -0.15, 0.40), VF-7 as -0.02 (95% CI: -0.15, 0.10), and Catquest Questionnaire as 1.45 (95% CI: -0.88, 2.01). The results for utility score, which were measured using various instruments, indicated non-significant improvement in the utility due to DSBCS compared to ISBCS. However, a significant improvement in post-operative utility score was seen using Catquest questionnaire for ISBCS compared to DSBCS. The included studies using VF-14 instrument were highly heterogeneous (I^2^ = 97.1%). Results provided SMD of -0.25 (95% CI:-1.06, 0.57) using VF-14 indicating non-significant improvement in the utility due to DSBCS compared to ISBCS surgery. Best corrected visual acuity (BCVA) significantly improved after both surgeries (overall SMD of BCVA due to ISBCS was -1.79 (95% CI: -2.45, -1.14) and due to DSBCS was -1.53 (95% CI: -2.25, -0.81)). A non-significant improvement was seen in BCVA due to ISBCS when compared to DSBCS (SMD = -0.18; 95% CI: -0.37, 0.01).

**Conclusion:**

Both surgeries, ISBCS and DSBCS significantly improve patients’ *quality of life* and visual acuity. Further, ISBCS may deliver certain additional benefits at the individual and societal levels as well.

## Introduction

According the World Health Organization, cataract is responsible for 48% of world blindness, a statistic that represents 18 million people [1]. Due to inadequate surgical services in developing countries, cataract remains a leading cause of blindness [2]. Even in developed countries where adequate surgical services are available, cataract may still remain prevalent [3] due to long wait times for operations. To enhance cataract surgical productivity, immediately sequential bilateral cataract surgery (ISBCS) [4]—cataract surgery that is performed on both eyes simultaneously—can deliver a plausible solution for patients needing surgery in both eyes [5] compared to delayed sequential bilateral cataract surgery (DSBCS), the surgery that is performed on each eye on a different day as a completely separate operation [6].

ISBCS provides significant benefits to patients, hospitals, and society [4]. The patients enjoy benefits in the form of fewer hospital visits, lack of anisometropia potentially reducing accidents and falls and one-step visual rehabilitation [4]. Lundstrom et al. concluded that even though visual rehabilitation is acquired to the same degree for DSBCS patients, ISBCS allowed rapid rehabilitation of the patients and helped avoid suboptimal visual function in daily life, which is a concern in the case of DSBCS [7,8]. Other advantages include shorter wait times for cataract surgery, more efficient use of operating room and clinic time, and more convenience for the patients’ support network (e.g., caregiver has to take less time off work) [4]. The hospital may benefit due to lower day-surgery-unit and costs [2].

As the traditional method for correcting cataracts, DSBCS offers the patient several advantages, including improved binocular visual acuity, contrast sensitivity and stereopsis [9]. Yet, benefits due to ISBCS exceed those of DSBCS [10,11]. The additional visit that is required for DSBCS may cause economic strain for the patient due to long waiting lists [2]. Between the two surgeries, patients may experience suboptimal visual function when performing daily life tasks [10]_._ Nassiri reported that anisometropia was detected in the DSBCS group but was non-existent in the ISBCS group [12]. Lundström et al. from Sweden indicated that DSBCS had a decreased value to patient compared to ISBCS, due to long waiting time for second-eye surgery and a short remaining lifetime [8]. Interestingly, another study showed DSBCS to be 48% more expensive compared to ISBCS [10]. There is also a 15% time savings due to ISBCS, which would lead to further savings [10]. Malvankar-Mehta et al. showed that ISBCS is a cost-effective procedure [11]. Overall, both surgeries provide an equal degree of long-term benefit to the patient, with both procedures offering similar results in terms of vision improvement [13].

In the literature, numerous studies have looked at the pros [9,14,15] and cons [16] of ISBCS and DSBCS. However, very few evaluate both surgeries, ISBCS and DSBCS [7,8,11,17–26].

Our research performs a systematic review and meta-analysis of ISBCS versus DSBCS, a project that comprises two main parts. In the first part, multiple bibliographic databases are systematically searched, and in the second part, meta-analysis is performed to compare ISBCS to DSBCS in order to answer two questions: 1) What are the quantitative effects on quality of life and visual outcomes of patients with cataract due to ISBCS compared to DSBCS? and 2) Are there differences in these quantitative effects for various kinds of bilateral cataract surgeries?

## Methods

### Search Strategy and Selection Criteria

In this work, we adhered to the Preferred Items for Systematic Reviews and Meta-Analyses (PRISMA) guidelines (S1 File). Literature, including published and unpublished scientific work, was systematically reviewed, and the following bibliographic databases were searched: MEDLINE (OVID and Pubmed), EMBASE (OVID), BIOSIS Previews (Thomson-Reuters), CINAHL (EBSCO), Health Economic Evaluations Database (HEED), ISI Web of Science (Thomson-Reuters) and the Cochrane Library (Wiley) from January 2000 to May 2014. Database specific subject headings and key words for “immediately sequential bilateral cataract surgery”, “simultaneous bilateral cataract surgery”, “delayed sequential bilateral cataract surgery”, “bilateral cataract”, and “cost of bilateral cataract surgery” were employed in the search strategy. The searches were modified to accommodate the unique terminology and syntax of each database (S2 File). Additionally, all synonyms were taken into account with the help of information specialist. Methodological filters were applied to limit our retrieval to economic studies, comparative studies, observational studies, meta-analyses, and systematic reviews. OVID AutoAlerts were set up to send monthly updates with any new literature. Monthly updates were performed on HEED, PubMed, and Cochrane Library databases.

Grey literature was identified by searching the conference abstracts of various meetings including the Canadian Ophthalmology Society meeting (COS), American Academy of Ophthalmology annual meeting (AAO), European Society of Ophthalmology (SOE), American Glaucoma society (AGS), and the Association for Research in Vision and Ophthalmology annual meeting (ARVO). The Proquest Dissertations and Theses databases and the Canadian Health Research Collection (Ebrary) were also searched for relevant content. Google and other internet search engines were used to search for additional web-based materials and information.

**Fig 1:**
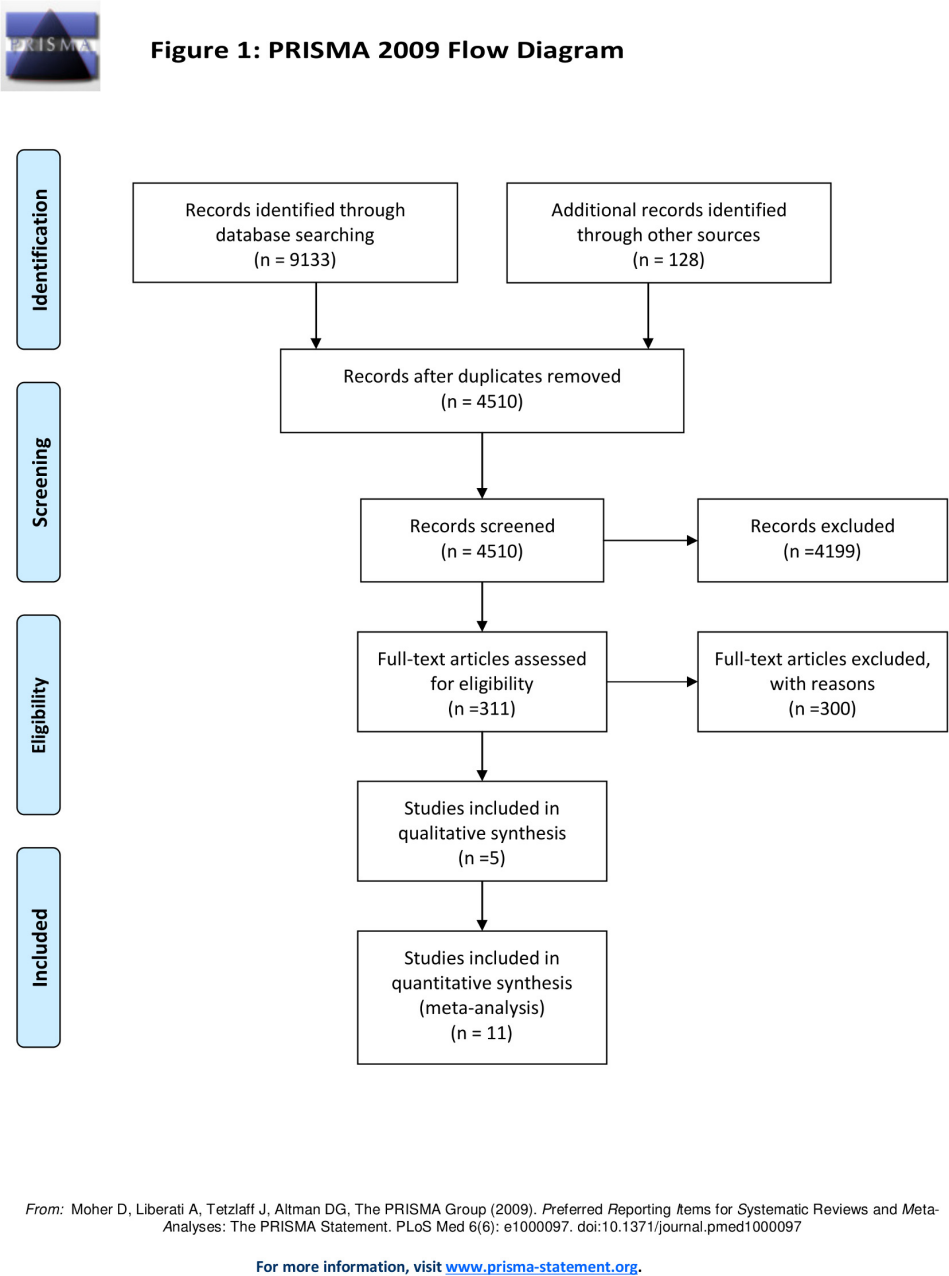
PRISMA Flow Diagram for Immediate versus Delayed Sequential Bilateral Cataract Surgery.

Inclusion criteria were: 1) publication in English language, 2) bilateral phacoemulsification on human subjects above the age of 19 and older, 3) publication dates from 2000 and onwards, 4) journal articles, systematic reviews, meta-analysis, cost analysis, cost-utility analysis, cost-effectiveness analysis, multicenter studies, randomized controlled trials, non-randomized studies including cohort studies (retrospective, prospective), clinical trials, and comparatives studies, and 5) studies conducted in North America, Europe, Australia, New Zealand, Japan, or Korea. These populations were selected since they represent relative homogeneity with respect to phacoemulsification procedures and services. Fig 1 summarizes the PRISMA Flow Diagram for including studies for meta-analysis.

In total, 9,133 records were retrieved from multiple databases and an additional 128 records were identified through grey literature search, conference proceedings, and hand-searches. These records were imported to EPPI-Reviewer 4 gateway (by EPPI-Centre, Social Science Research Unit, the Institute of Education, University of London, UK) to remove duplicates. After removing duplicates, 4,510 records were included for the three-level screening process. Level 1 screening involved reviewing titles, while Level 2 screening reviewed abstracts, and Level 3 screening involved full-text reviews of each included study by two reviewers, independently (S4). At each level, agreement for inclusion between the two reviewers was assessed by Cohen’s kappa (κ) coefficient. Articles were included for the next level of screening if both the reviewers agreed. Differences between the reviewers were discussed and resolved by consensus. In cases where consensus was not achieved, a third reviewer was brought in to provide a decision.

After conducting title, abstract, and full-text screening, 311 records were assessed for eligibility. From an initial total of 11 eligible studies, two studies by the same author (Busbee et al. 2002 [19] and 2003[20]) were consolidated, resulting in a final total of 10 studies that were eligible for meta-analysis (Table 1). From these 10, nine studies stated the data on pre-operative and post-operative utility score using various instruments (Table 2), all studies reported the data on pre-operative and post-operative best-corrected visual acuity (Table 3), and five studies listed the complications that occurred from both types of surgeries (Table 4). In the end, a total of 10 studies were included for quantitative synthesis and five studies for qualitative analysis (Fig 1).

**Table 1:** Charaterisctics of Studies Included in Meta-Analysis.

Author (Year)	Study Design	Study Location	Procedure	N	MeanAge	Age(SD)
Busbee (2002[19 ^]^, 2003[20])	CEA	U.S.	ISBCS	486	73	0
	CEA	U.S.	DSBCS	486	73	0
Castells (2006)[21]	RCT	Spain	ISBCS	139	71.7	9.07
	RCT	Spain	DSBCS	135	72.03	8.87
Chung (2009)[17]	Cohort	New Zealand/ Australia/Japan	ISBCS	94	66.38	9.41
	Cohort	New Zealand/ Australia/Japan	DSBCS	100	65.32	11.11
Gothwal (2011)[18]	Cohort	New Zealand/ Australia/Japan	ISBCS	29	72.9	9.8
	Cohort	New Zealand/ Australia/Japan	DSBCS	38	72.9	9.8
Hiratsuka (2011)[22]	CEA	Japan	ISBCS	312	72.2	7.7
	CEA	Japan	DSBCS	60	69.9	7.9
Leivo (2011)[23]	CEA	Finland	ISBCS	250	75.3	7.9
	CEA	Finland	DSBCS	257	75	8.1
Lundstrom (2006)[7]	RCT	Sweden	ISBCS	50	72.5	0
	RCT	Sweden	DSBCS	46	72.5	0
Lundstrom (2009)[8]	Cohort	Sweden	ISBCS	17	77.9	9
	Cohort	Sweden	DSBCS	80	77.9	9
Sarikkola (2011)[25]	RCT	Finland	ISBCS	250	75.3	7.9
	RCT	Finland	DSBCS	257	75	8.1
Serrano-Aguilar (2012)[24]	RCT	Spain	ISBCS	439	72.9	8.2
	RCT	Spain	DSBCS	406	71.7	7.9

N = sample size, SD = standard deviation, CEA = cost-effectiveness analysis, RCT = randomized control trial, ISBCS = immediately sequential bilateral cataract surgery, DSBCS = delayed sequential bilateral cataract surgery.

**Table 2:** Utility Scores Reported in Studies Included in Meta-Analysis.

Author(Year)	Procedure	Pre-operative utility score(Mean)	Pre-operative utility score(SD)	Post-operative utility score (Mean)	Post-operative utility score(SD)	Instrument used
Busbee (2002[19], 2003[20])	ISBCS	-	-	0.967	0.0	TTO
	DSBCS	-	-	0.858	0.0	TTO
Castells (2006)[21]	ISBCS	58.08	20.59	97.7	7.1	VF-14
	DSBCS	61.01	22.28	89.5	15.9	VF-14
Hiratsuka (2011)[22]	ISBCS	0.58	0.29	0.85	0.25	TTO
	ISBCS	0.84	0.15	0.9	0.15	EQ5D
	ISBCS	0.62	0.24	0.76	0.25	HUI3
	DSBCS	0.64	0.29	0.88	0.23	TTO
	DSBCS	0.83	0.16	0.92	0.13	EQ5D
	DSBCS	0.7	0.2	0.79	0.22	HUI3
Leivo (2011)[23]	ISBCS	65.5	18.1	24.3	21.0	VF-7
	DSBCS	65.6	19.6	23.8	19.2	VF-7
Lundstrom (2006)[7]	ISBCS	13.5	21.0	7.0	0.0	Catquest Questionnaire
	DSBCS	13.0	21.0	7.0	0.0	Catquest Questionnaire
Lundstrom (2009)[8]	ISBCS	13.5	21.0	8.0	2.075	Catquest Questionnaire
	DSBCS	13.0	21.0	11.0	2.075	Catquest Questionnaire
Malvankar-Mehta (2013)[11]	ISBCS	-	-	0.97	0.0	TTO
	DSBCS	-	-	0.89	0.0	TTO
Sarikkola (2011)[25]	ISBCS	65.5	18.1	24.3	21.0	VF-7
	DSBCS	65.6	19.6	23.8	19.2	VF-7
Serrano-Aguilar (2012)[24]	ISBCS	66.6	22.7	95.3	11.0	VF-14
	DSBCS	66	21.4	96.9	8.5	VF-14

QOL = quality of life, TTO = time trade-off [35], VF-7 = visual function questionnaire—7 [36], VF-14 = visual function questionnaire—14 [36,37], EQ5D = EuroQOL five dimensions questionnaire [38,39], HUI3 = health utility index mark 3 [40].

**Table 3:** Best Corrected Visual Acuity (BCVA) Reported in Studies Included in Meta-Analysis.

Author (Year)	Procedure	Pre-operative BCVA (logMAR)(Mean)	Pre-operative BCVA (logMAR)(SD)	Post-operative BCVA (logMAR)(Mean)	Post-operative BCVA (logMAR)(SD)
Busbee (2002[19], 2003[20])	ISBCS	0.618	0	0.1303	0
	DSBCS	0.618	0	0.1303	0
Castells (2006)[21]	ISBCS	0.54	0.17	0.11	0.1
	DSBCS	0.56	0.19	0.18	0.17
Chung (2009)[17]	ISBCS	0.31	0.17	0.11	0.12
	DSBCS	0.29	0.16	0.1	0.11
Gothwal (2011)[18]	ISBCS	0.42	0.26	0.2	0.23
	DSBCS	0.4	0.35	0.19	0.27
Hiratsuka (2011)[22]	ISBCS	0.51	0.52	0.03	0.25
	DSBCS	0.51	0.52	0.03	0.25
Leivo (2011)[23]	ISBCS	0.477	0.176	0.0969	0.175
	DSBCS	0.477	0.176	0.176	0.175
Lundstrom (2006)[7]	ISBCS	0.2	0	0	1.6
	DSBCS	0.2	0	0.1	1.6
Lundstrom (2009)[8]	ISBCS	0.2	1.7	0	1.52
	DSBCS	0.2	1.7	0	1.52
Sarikkola (2011)[25]	ISBCS	0.477	0.175	0.1	0.195
	DSBCS	0.477	0.175	0.2	0.195
Serrano-Aguilar (2012)[24]	ISBCS	0.699	0.2	0.0414	0.2
	DSBCS	0.699	0.176	0.0414	0.2

BCVA = best corrected visual acuity, logMAR = Logarithm of the Minimum Angle of Resolution

**Table 4:** List of Complications Reported in Included Studies.

Author(Year)	Complications after ISBCS (rate in %)	Complications after DSBCS (rate in %)
Chung (2009)[17]	Uveitis (0.53), posterior capsule rupture (1.06), transient IOP spike (2.13)	Posterior capsule rupture (1), transient IOP spike (2.5)
Lundstrom (2006)[7]	High IOP (2), corneal edema (1), post-operative iritis (1), vitreous detachment (1), posterior capsule opacification (2)	High IOP (2), corneal edema (1), post-operative iritis (1), vitreous detachment (1), posterior capsule opacification (2)
Sarikkola (2011)[25]	CME (0.2), anterior capsule tear (1.8), posterior capsule tear (2), zonular tear (0.2), vitreous loss (1.2), sphincterotomy (1.6), sutures in wound (2.4), IOP >30mm Hg (10.6), wound leak (0.2), out-of-bag IOL implantation (1.2), central corneal edema (7.4), IOL decentration, anterior (0.6), chamber flare (2.2)	CME (0.8), posterior capsule fibrosis (6.6), anterior capsule tear (0.8), posterior capsule tear (2.6), zonular tear (0.8), vitreous loss (1.4), sphincterotomy (0.6), sutures in wound (4.8), IOP >30mm Hg (13.8), wound leak (0.6), out-of-bag IOL implantation (1.4),
Serrano-Aguilar (2012)[24]	Central corneal edema (5.9), IOL decentration (0.8), anterior chamber flare (1.2)	Posterior capsule fibrosis (7.7), minor posterior capsule opacification (0.12), posterior capsule tear (0.13), immediate corneal edema (0.38), foreign-body sensation (0.13)

ISBCS: immediately sequential bilateral cataract surgery, DSBCS: delayed sequential bilateral cataract surgery, IOP: intraocular pressure, CME: cystoid macular edema, IOL: intraocular lense

### Data Extraction

Data was extracted from the 11 eligible articles using a data extraction form. First reviewer extracted data from the included studies, and a second reviewer resolved errors by reviewing the extracted data. Data extracted included study objective, study design, location setting, inclusion and exclusion criteria, data collection technique, data collection period, total patients enrolled in the study, total patients enrolled in and completed the study, refusal to consent, number of females, patient demographic characteristics, follow-up, time between surgeries, quality of life (QOL) outcomes (including utility, visual disability score, vision QOL score), and baseline and post-operative characteristics including (bilateral visual acuity corrected (BCVA), contrast sensitivity, refractive error, stereopsis). For missing data, various pieces of available information (such as the range, *p*-value, and confidence interval) were utilized and converted to the common effect measure–SD. Further, corresponding authors were also contacted for additional information. Using a Downs and Black checklist [27], each included article was independently appraised by the two reviewers for quality.

Our main findings of the systematic review have been summarized in a tabular form. Table 1 lists the characteristics of the extracted studies, including study design, study location, procedure, sample size, and age. Table 2 lists the utility scores of the extracted studies, including pre-operative and post-operative utility scores, as well as the instrument used to collect the utility data. A total of nine studies provided the utility data. Specifically, one study from Japan compared utility values for both the surgeries using three different instruments: a time-trade off (TTO) questionnaire; a Euro quality-of-life, five-dimensional (EQ5D) questionnaire; and a Health Utilities Index Mark 3 (HUI3)[22]. Two studies from the same author used a Catquest Questionnaire to obtain pre-operative and post-operative utility data [7,8], while four European studies used the visual function (VF) questionnaire to obtain QOL data. Table 3 lists the pre-operative and post-operative BCVA of the extracted studies. Table 4 lists post-operative complications, as well as the rate of complications for both types of surgeries.

### Statistical Analysis

Meta-analysis was utilized to combine findings from several independent studies that attempted to evaluate the benefits of ISBCS compared to DSBCS. By statistically combining the findings such as pre- and post-operative utility score and pre- and post-operative BCVA from these independent studies, the power of the analysis increased considerably, resulting in a single-effect estimate of utility score and BCVA called the summary effect.

STATA v. 13.0 (STATA Corporation, College Station, TX) was used to conduct meta-analysis. Standardized mean difference (SMD) was computed as the effect size. SMD of the mean pre- and post-operative utility score was chosen as the effect size to separately evaluate the efficacy of both types of surgeries. Additionally, the SMD of the mean post-operative utility score due to ISBCS and DSBCS was chosen as the effect size to evaluate the efficacy of ISBCS versus DSBCS. The SMD was stratified based on the use of an instrument such as TTO, VF-14, EQ5D questionnaire, HUI3, VF-7 questionnaire, and Catquest Questionnaire to calculate the utility score due to ISBCS and DSBCS and for ISBCS versus DSBCS. To explore the effect of each surgery separately, as well as to identify the combined effect on utility, studies were pooled using the fixed-effect model. To test heterogeneity, I^2^ statistics was computed and χ^2^ test [28] was performed. In case of significant heterogeneity, that is, a low p-value and large χ^2^ statistics and I^2^ statistics [29], analysis was redone to compute random-effect model using DerSimonian and Laird method. Forest plots were also generated.

The SMD of the mean pre- and post-operative BCVA in logMAR units of ISBCS and DSBCS was chosen as the effect size to separately evaluate the efficacy of both types of surgeries. Additionally, the SMD of the mean post-operative BCVA in logMAR units due to ISBCS and DSBCS was chosen as the effect size to evaluate the efficacy of ISBCS versus DSBCS. To explore the effect of each surgery separately and to identify the combined effect of ISBCS versus DSBCS on BCVA, studies were pooled using the fixed-effect model. A *Z*-value to test the null hypothesis was conducted. Heterogeneity was determined using the I^2^ value and a χ^2^ test. Forest plots were generated. Due to evidence of heterogeneity, a random-effect model was computed.

Funnel plot was used to assess the risk of publication bias. Funnel plots for each sub-group, TTO, EQ5D, HUI3, VF-7, Catquest questionnaire, and VF-14 were not considered since just one study reported utility score by TTO, EQ5D, and HUI3 and two studies reported utility score by VF-14, VF-14, and Catquest Questionnaire.

## Results

### Study Characteristics

The characteristics of the studies involved in meta-analysis included: datasets from the United States [19,20], Spain [21,24], Finland [23,25], Sweden [7,8], New Zealand [17,18], Australia [17,18], and Japan [17,18,22]. Further, the study design of the included works examined cost-effectiveness analysis (CEA) [11,19,20,22,23], cohort studies [17,18], and randomized control trials (RCT) [7,21,24,25]. The follow-up after both the surgeries ranged from one day to one year. For DSBCS, the time between the first eye surgery and the second eye surgery ranged from two days to six months.

### Publication Bias

**Fig 2:**
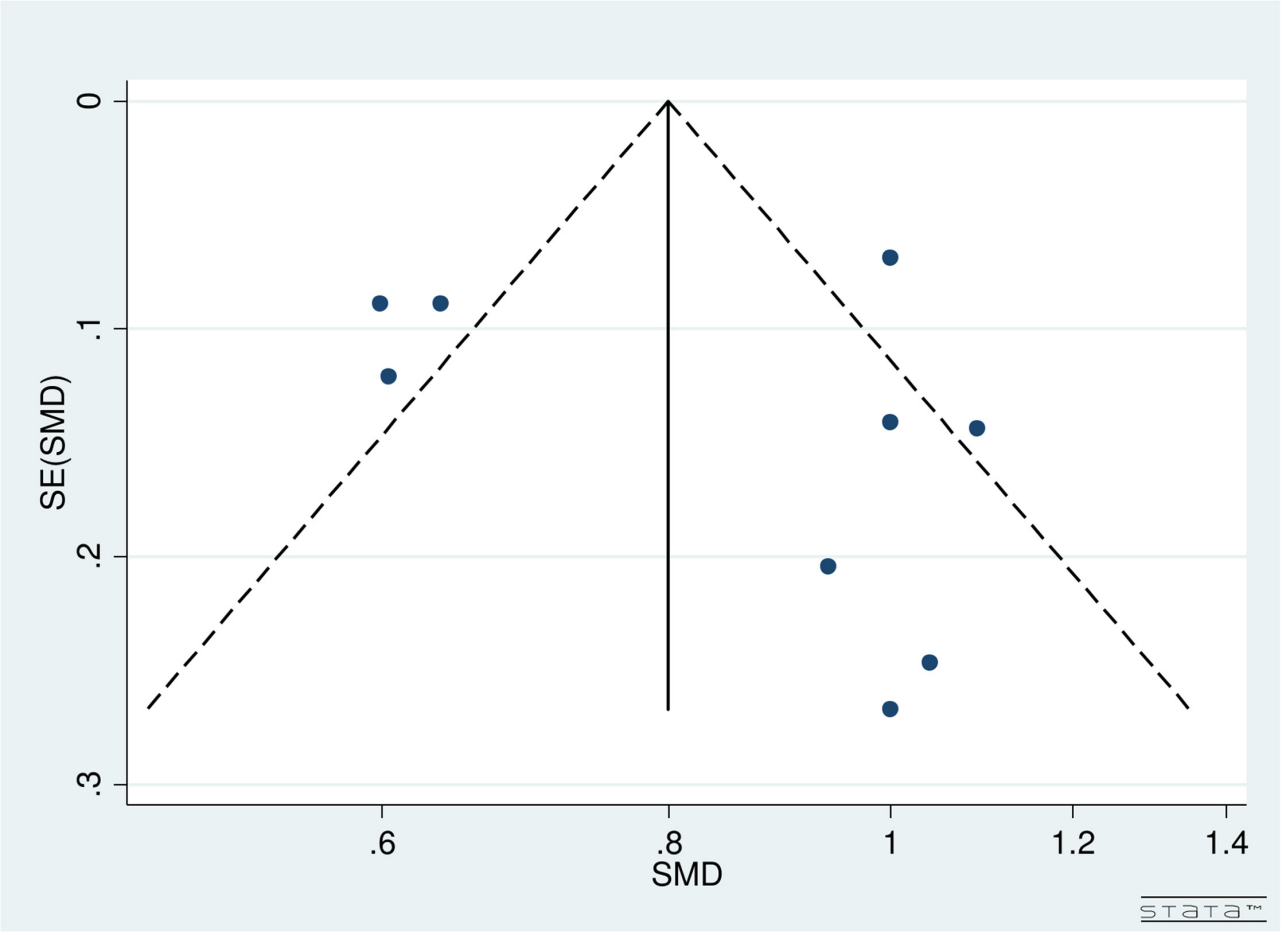
Funnel Plot for Included Studies Evaulating Immediate versus Delayed Sequential Bilateral Cataract Surgery.

In Fig 2, funnel plot for studies evaluating both the surgeries was not fully symmetrical. The included studies were scattered from top to bottom right and top left corner of the plot. Therefore, publication bias could not be concluded. Partially, the reason was difficulty in interpretation of funnel plot for a small group of studies, high heterogeneity (see below) and small effect sizes. Additionally, publication bias is only one of the numerous possible explanations for funnel plot asymmetry.

### Effect on Utility

**Fig 3:**
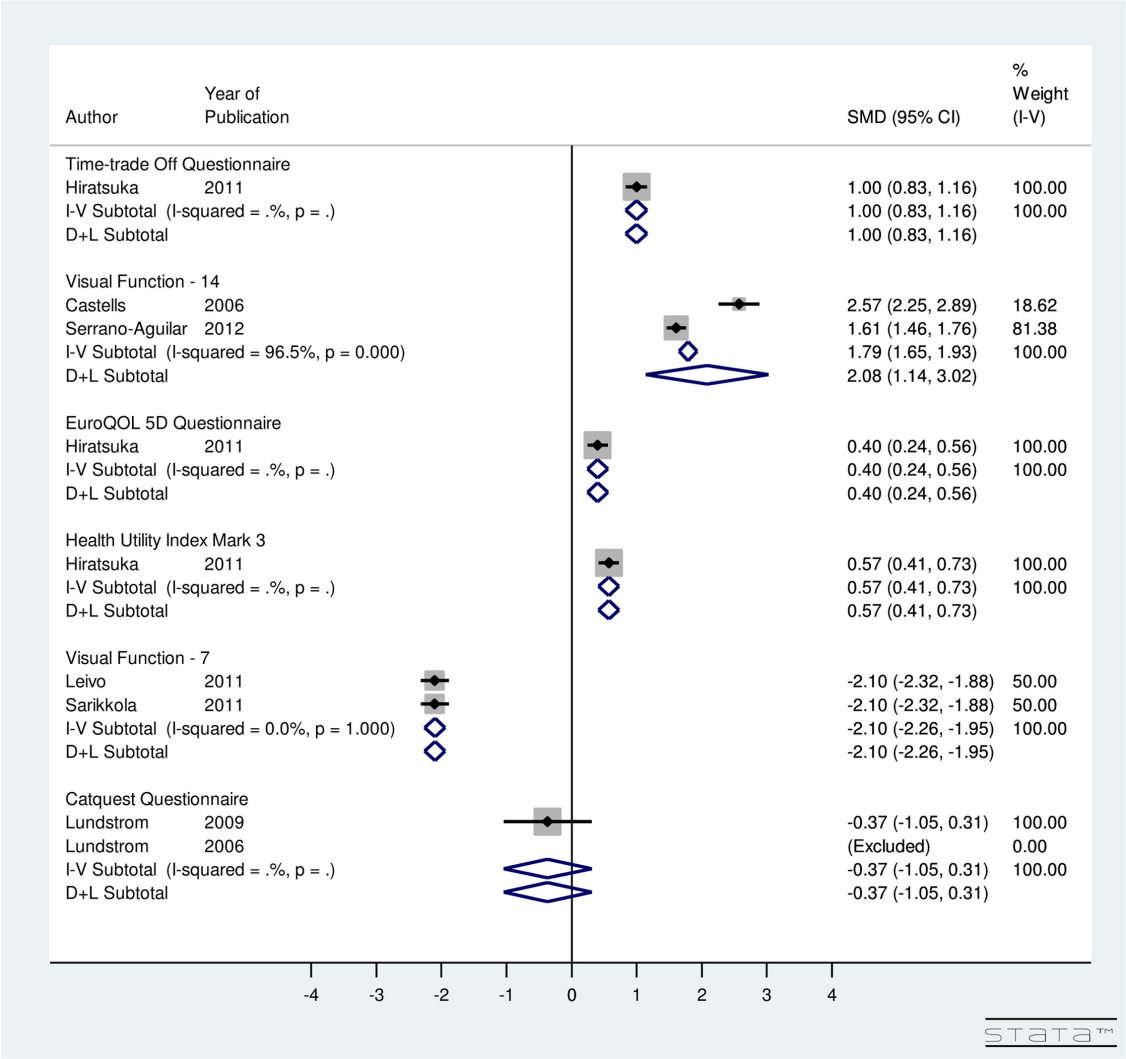
Forest Plot showing Improvement in Utility Due to Immediately Sequential Bilateral Cataract Surgery.

In Fig 3, meta-analysis is done by instrument used to calculate utility score. For ISBCS, utility-specific fixed-effect computations provided an overall SMD of the utility score using the TTO method as 1.00 (95% CI: 0.83, 1.16), EQ5D as 0.40 (95% CI: 0.24, 0.56), HUI3 as 0.57 (95% CI: 0.41, 0.73), VF-7 as -2.10 (95% CI: -2.26, -1.95), and Catquest questionnaire as -0.37 (95% CI: -1.05, 0.31) (Fig 3). The results indicate that post-operative utility score using TTO, EQ5D, HUI3, and VF-7 does improve significantly for ISBCS. A non-significant improvement in post-operative utility score is seen using Catquest questionnaire for ISBCS surgery. Regarding the VF-14 score, the included studies were highly heterogeneous (I^2^ = 96.5%). Random-effect model provided an overall SMD of 2.08 (95% CI: 1.14, 3.02) using VF-14. This finding implies that the VF-14 score significantly improves due to ISBCS.

**Fig 4:**
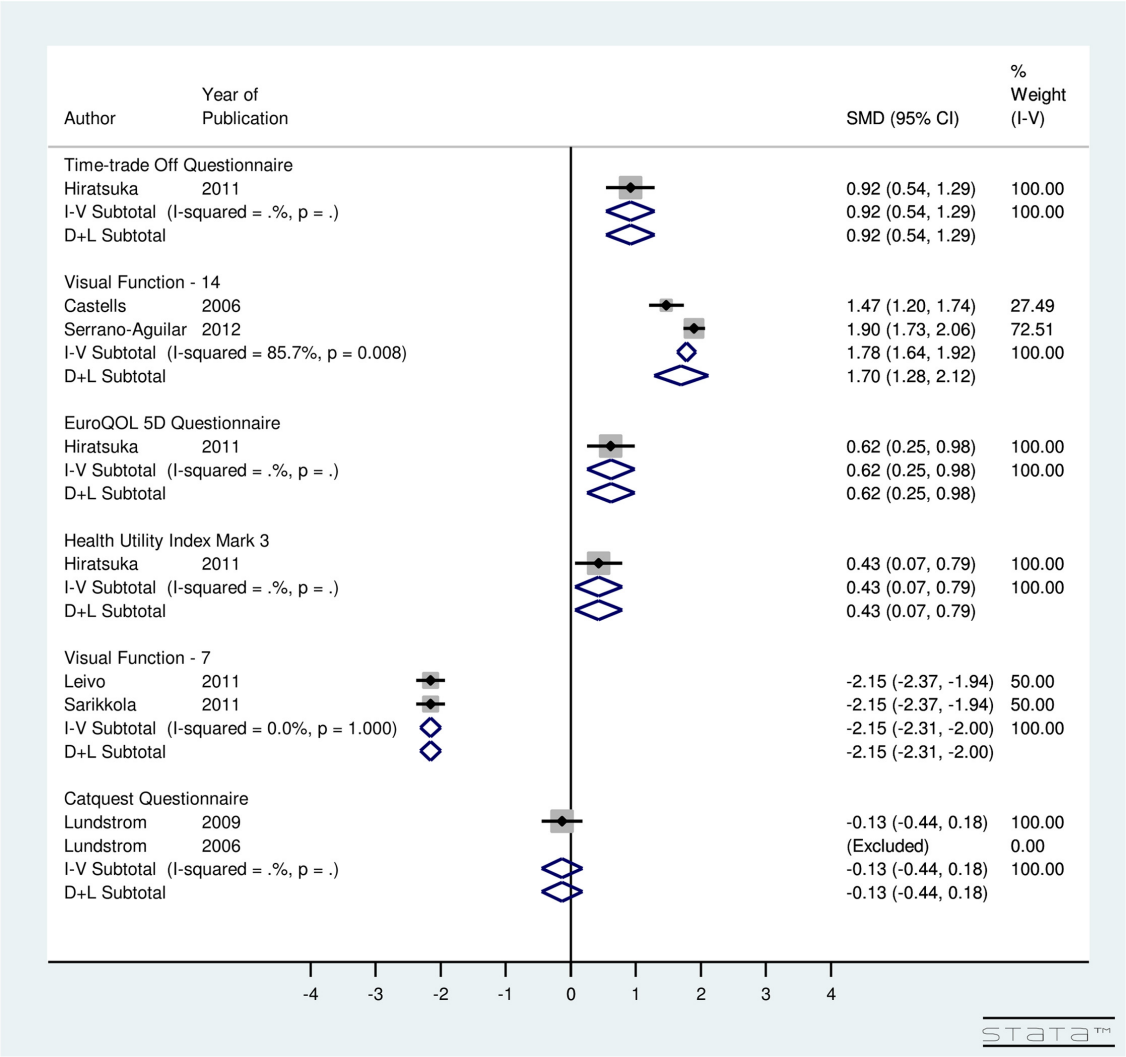
Forest Plot showing Improvement in Utility Due to Delayed Sequential Bilateral Cataract Surgery.

For DSBCS, utility-specific fixed-effect meta-analysis provided an overall SMD of 0.92 (95% CI: 0.54, 1.29) using TTO, 0.62 (95% CI: 0.25, 0.98) using EQ5D, 0.43 (95% CI: 0.07, 0.79) using HUI3, -2.15 (95% CI: -2.31, -2.0) using VF-7, and -0.13 (95% CI: -0.44, 0.18) using Catquest Questionnaire (Fig 4). The results indicate that post-operative utility score using TTO, EQ5D, HUI3, AND VF-7 does improve significantly for DSBCS. A non-significant improvement in post-operative utility score is seen using Catquest questionnaire for DSBCS surgery. Regarding the VF-14 score, the included studies were highly heterogeneous (I^2^ = 85.7%) and random-effect computation provided SMD of 1.7 (95% CI: 1.28, 2.12) using VF-14. This finding suggests that the VF-14 score improves significantly due to DSBCS.

**Fig 5:**
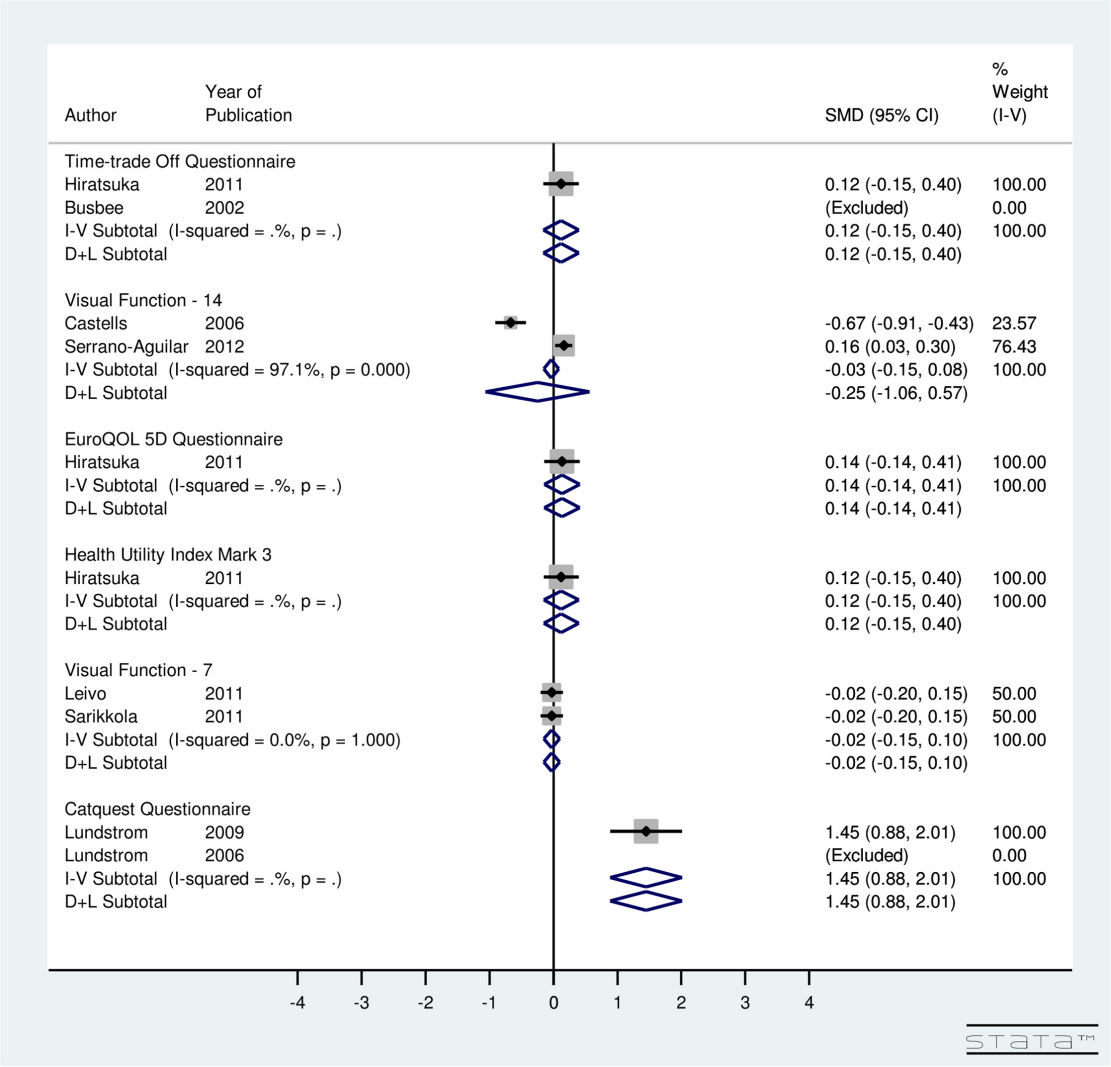
Forest Plot showing Improvement in Utility Due to Immediately Sequential Bilateral Cataract Surgery versus Delayed Sequential Bilateral Cataract Surgery.

For ISBCS versus DSBCS, utility-specific fixed-effect model provided an overall SMD of the utility score using the TTO method as 0.12 (95% CI: -0.15, 0.40), EQ5D as 0.14 (95% CI: -0.14, 0.41), HUI3 as 0.12 (95% CI: -0.15, 0.40), VF-7 as -0.02 (95% CI: -0.15, 0.10), and Catquest Questionnaire as 1.45 (95% CI: -0.88, 2.01) (Fig 5). The results for utility score, which were measured using various instruments, indicated non-significant improvement in the utility due to ISBCS compared to DSBCS. However, a significant improvement in post-operative utility score is seen using Catquest questionnaire for ISBCS compared to DSBCS. The included studies using VF-14 instrument were highly heterogeneous (I^2^ = 97.1%). Results provided SMD of -0.25 (95% CI:-1.06, 0.57) using VF-14 indicating non-significant improvement in the utility due to DSBCS compared to ISBCS surgery.

### Effect on Best Corrected Visual Acuity (BCVA)

**Fig 6:**
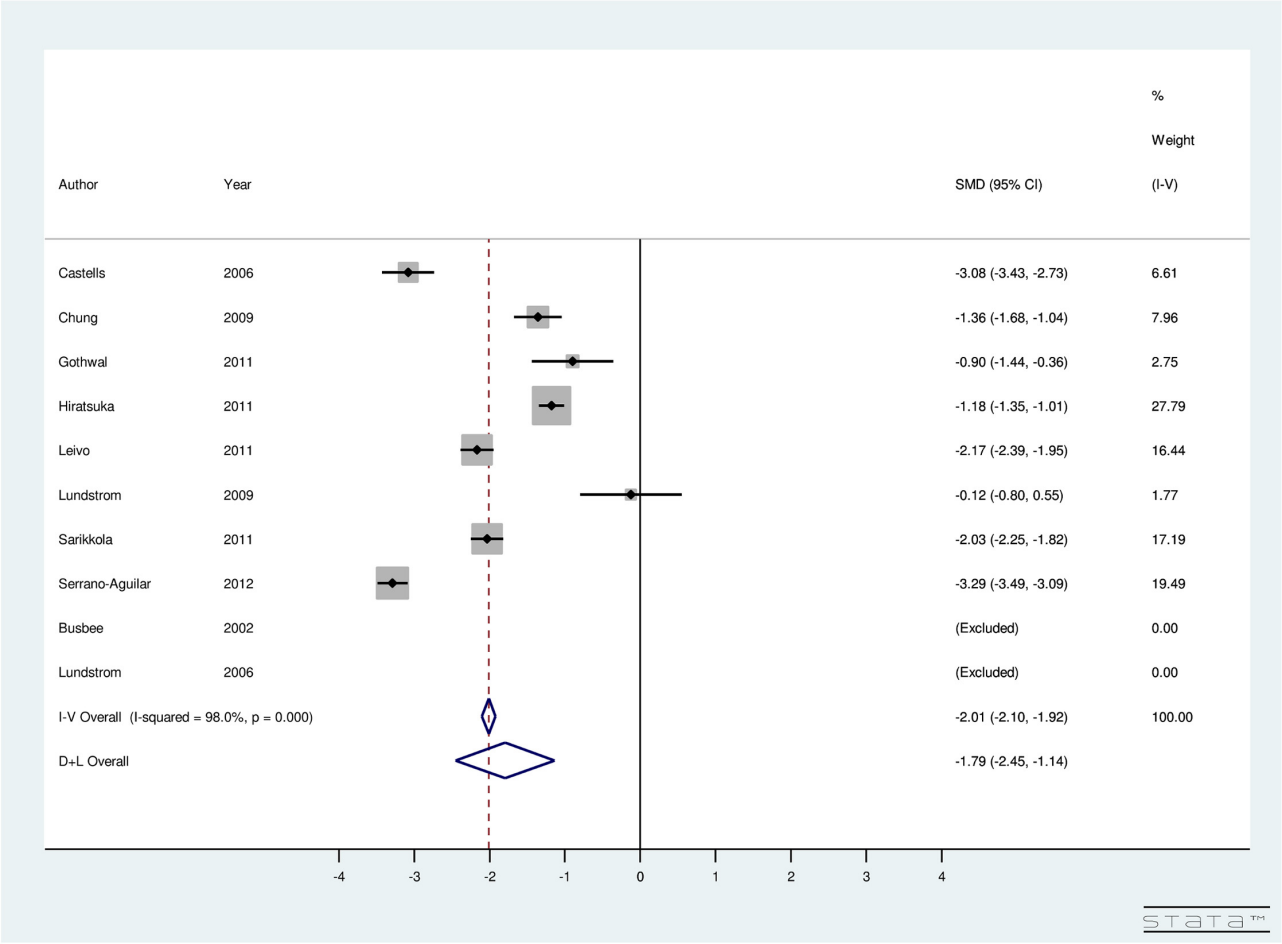
Forest Plot showing Improvement in Best Corrected Visual Acuity Due to Immediately Sequential Bilateral Cataract Surgery.

**Fig 7:**
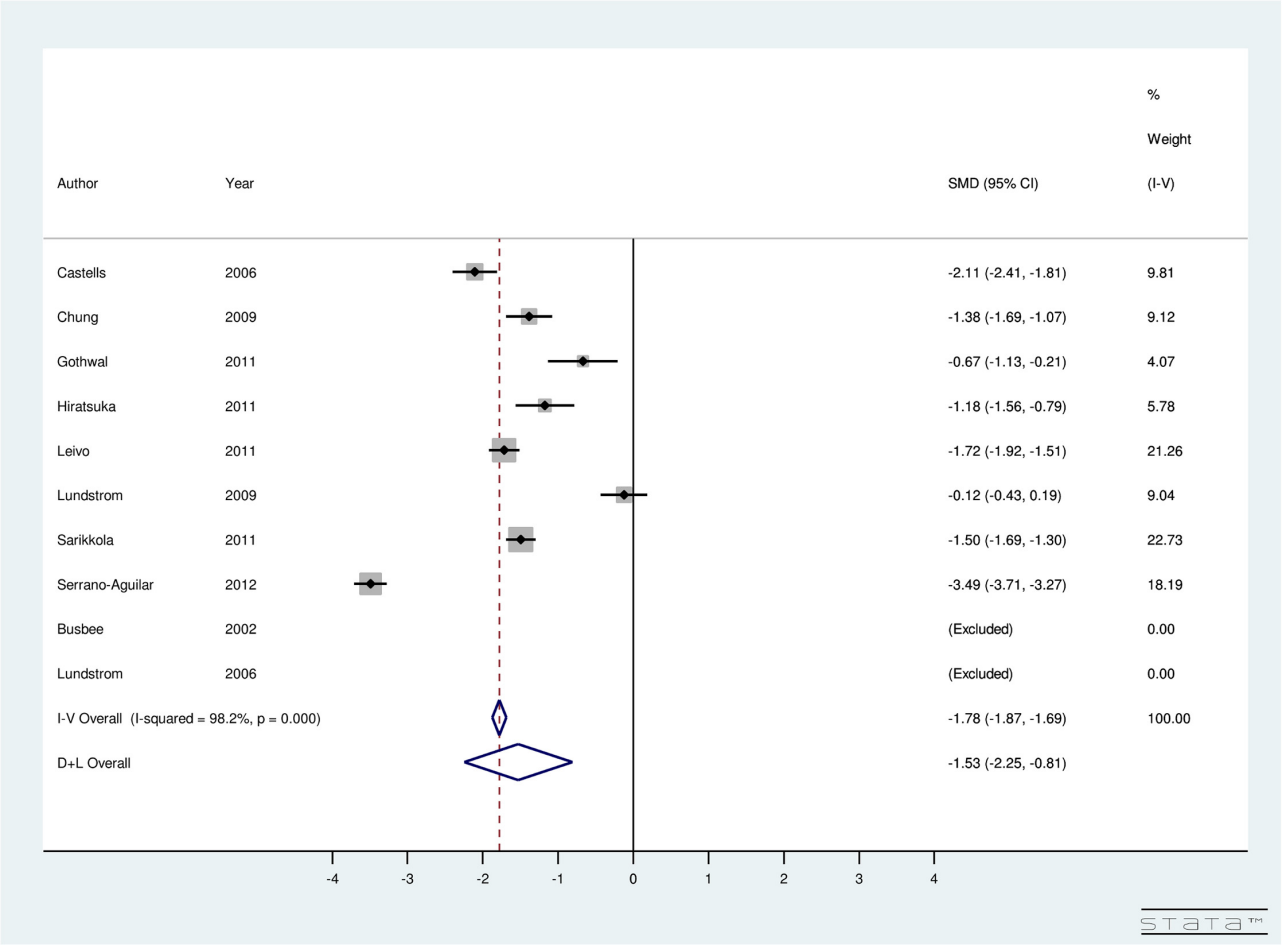
Forest Plot showing Improvement in Best Corrected Visual Acuity Due to Delayed Sequential Bilateral Cataract Surgery.

Our results indicate improvement in BCVA after both surgeries (See Figs 6 and 7 for details). This result is consistent with the published literature on ISBCS and DSBCS. The studies included in the meta-analysis were highly heterogeneous (I^2^ = 98% for ISBCS analysis and I^2^ = 98.2% for DSBCS analysis). Therefore, a random-effect meta-analysis was conducted to provide an overall SMD of BCVA due to ISBCS of -1.79 (95% CI: -2.45, -1.14) and due to DSBCS of -1.53 (95% CI: -2.25, -0.81). This result suggests significant improvement in BCVA after both surgeries.

**Fig 8:**
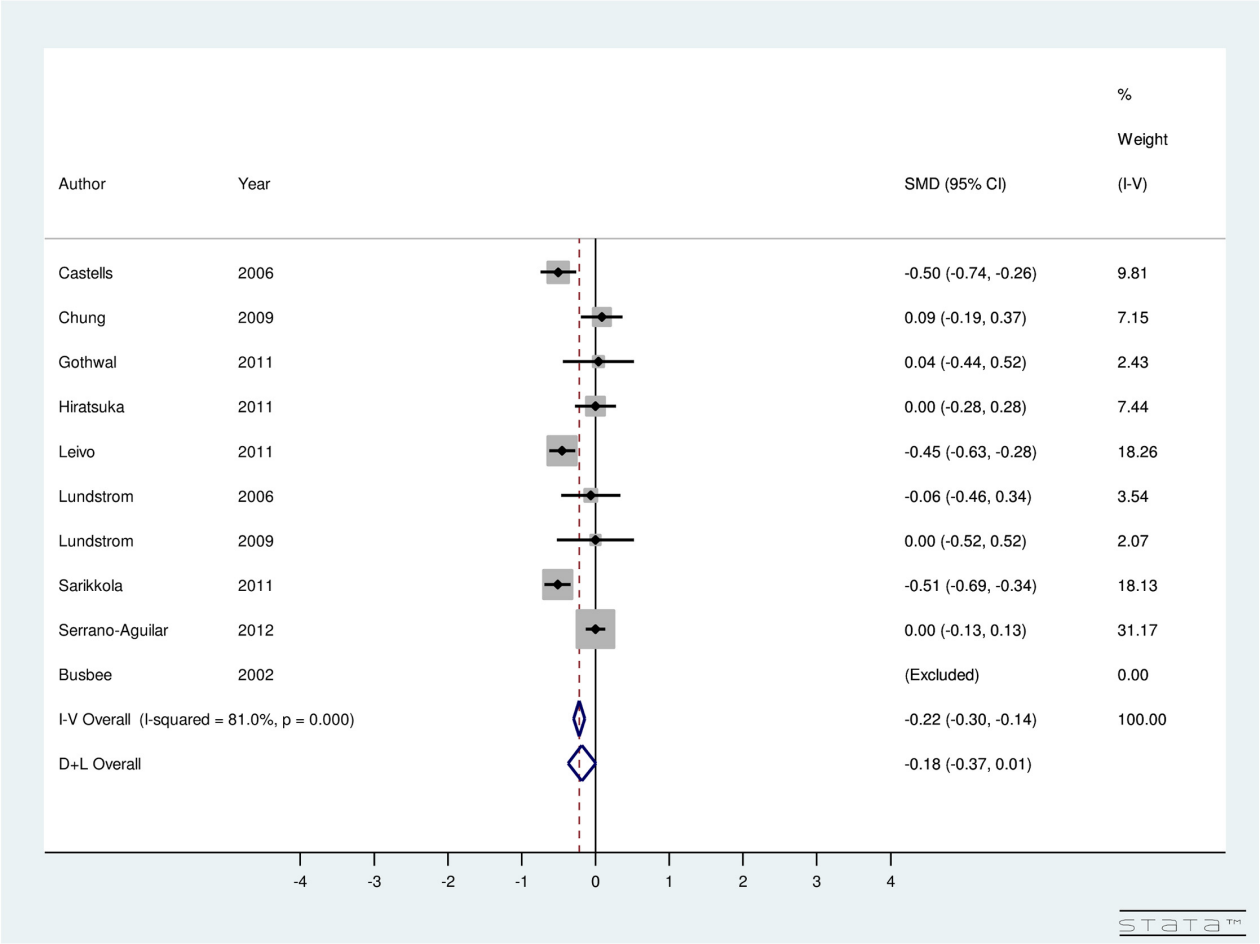
Forest Plot showing Improvement in Best Corrected Visual Acuity Due to Immediately Sequential Bilateral Cataract Surgery versus Delayed Sequential Bilateral Cataract Surgery.

Additionally, from Fig 8, a non-significant improvement is seen in BCVA due to ISBCS when compared to DSBCS. The studies included were highly heterogeneous (I^2^ = 81%). Random-effect computations showed an overall SMD of BCVA of ISBCS and DSBCS as -0.18 (95% CI: -0.37, 0.01). This finding suggests that improvement in BCVA after both surgeries is almost similar.

## Discussion

During the systematic review, 9,133 research articles, conference proceedings, abstracts, and grey literature were systematically reviewed after searching various bibliographic databases. Eleven articles with 3,657 subjects were included for quantitative analysis. Meta-analysis of ISBCS versus DSBCS was conducted by aggregating the results to obtain a common-effect estimate.

Our results indicated that post-operative utility score due to both the surgeries–ISBCS and DSBCS–does improve. Both surgeries provide an equal degree of improvement in utility score or patient’s quality of life. Further, the post-operative BCVA due to both surgeries improved. Nonetheless, both procedures provide similar level of improvement in vision. A significant strength of our analysis stems from the fact that all the included studies had coherent results of improvement in utility score or patient’s quality of life after both types of surgeries and due to ISBCS compared to DSBCS. Similar coherent results were seen in all identified studies for BCVA.

Given the high heterogeneity between studies, a random-effect model was developed to calculate the overall SMD. This substantial degree of heterogeneity could be a consequence of several factors, including consistency in the way the procedures/surgeries were performed, geographic location, available facilities to perform surgeries, follow-up period, time between surgeries, rates of complications, year the surgeries were performed, year the study was conducted, etc. On the other hand, our purpose was to quantitatively test the hypotheses on the source of heterogeneity.

Further, our study contains certain limitations. First, the quality of the included studies was accessed using a Downs and Black checklist [27], and we did find high-, medium-, and poor-quality studies. However, we included all 13 studies, irrespective of their quality, because of the limited number of articles we encountered. Second, we found only limited information on pre-operative and post-operative contrast sensitivity, spherical equivalent or refraction difference, stereopsis, and astigmatism with which to conduct our quantitative analysis, and thus, these characteristics had to be excluded from the analysis. Third, meta-analysis of observational studies is influenced by inherent biases in the included articles [28]. For example, a multitude of other factors–income status, socioeconomic status, previous ocular and non-ocular surgeries, family history, other ocular and non-ocular diseases, pre-operative and post-operative medications, number of medications, comorbidities (e.g., high blood pressure, diabetes, stroke, heart conditions, etc.)–can influence the estimates in the original studies. Fourth, many studies had to be excluded due to a lack of necessary information. If all the excluded studies had been considered, our results might well have been influenced. Such an inclusion would be unlikely to make a considerable impact, however, since our quantitative analysis agreed with published meta-analysis.

In sum, our results showed that both delayed sequential bilateral cataract surgery (DSBCS) and immediately sequential bilateral cataract surgery (ISBCS) significantly improves patients’ quality of life and visual acuity. Further, ISBCS may deliver certain additional benefits at the individual and societal levels as well. A substantial amount of research has showed declining rates of a major post-operative complications such as endophthalmitis [16,26,30–32] and cystoid macular edema (CME) [33,34] due to ISBCS and no case of bilateral endophthalmitis has been observed when careful sterile separation is considered. And finally, little attention has been paid to the process of identifying numerous factors (e.g., previous ocular and non-ocular surgeries, family history, other ocular and non-ocular diseases, pre-operative and post-operative medications, number of medications, comorbidities) that can influence the estimates due to both types of surgeries.

To conclude, using meta-analysis, both surgeries provide an equal degree of benefit to the patient, with both procedures offering similar results in terms of patient’s quality-of-life and vision improvement. ISBCS has additional benefits at the individual as well as societal levels.

## Supporting Information

S1 File PRISMA 2009 Checklist. (DOC)

S2 File Search Strategy for EMBASE. (DOCX)

S3 File Level 1, 2, and 3 screening questions. (DOCX)
