# Supplementary material for: Immediate versus Delayed Sequential Bilateral Cataract Surgery: A Systematic Review and Meta-Analysis
Source: PLoS One. 2015 Jun 29;10(6):e0131857. doi: 10.1371/journal.pone.0131857 (PMC4485471; doi:10.1371/journal.pone.0131857)
Supplement: S2 File — (DOCX) [file pone.0131857.s002.docx]

**S2: Search strategy for EMBASE**

Database: Embase Classic + Embase <1947 to 2014 March 28>

Search Strategy:
--------------------------------------------------------------------------------
1     Phacoemulsification/ (9442)
2     (phaco* or phako* or Emulsification, Len* or Lens emulsification).mp. (14193)
3     1 or 2 (14193)
4     (Bilateral or two* or both* or sequential or immediate* delayed* or simult* or delayed* or same day or different day).mp. (7349426)
5     3 and 4 (4738)
6     (delayed sequential bilateral cataract* or delayed bilateral cataract* or DSBCS or different day sequential bilateral cataract* or different-day sequential bilateral cataract* or different day cataract* or bilateral different day cataract* or bilateral different-day cataract* or delayed sequential bilateral pha* or delayed bilateral pha* or different day sequential bilateral pha* or different-day sequential bilateral pha* or different day pha* or bilateral different day pha* or bilateral different-day pha*).mp. (13)
7     (immediate sequential bilateral cataract* or immediately sequential bilateral cataract* or immediate bilateral cataract* or immediately bilateral cataract* or ISBCS or simultaneous bilateral cataract* or same day sequential bilateral cataract* or same-day sequential bilateral cataract* or same day cataract* or bilateral same day cataract* or bilateral same-day cataract* or immediate sequential bilateral pha* or immediately sequential bilateral pha* or immediately bilateral pha* or immediate bilateral pha* or simultaneous bilateral pha* or same day sequential bilateral pha* or same-day sequential bilateral pha* or same day pha* or bilateral same day pha* or bilateral same-day pha*).mp. (90)
8     5 or 6 or 7 (4812)
9     exp animals/ or invitro/ or (animal or rat$ or mouse or mice or "in vitro").mp. (21039935)
10     human/ or men/ or women/ or male/ or female/ or (man or men or woman or women or male$ or female$ or human).mp. (17270136)
11     Adolescent/ or exp Child/ or exp Infant/ or adolescen$.mp. or child$.mp. or [infant.mp](http://infant.mp/). or teenage$.mp. (3283033)
12     exp Adult/ or adult$.mp. or [aged.mp](http://aged.mp/). or elder$.mp. or middle [age.mp](http://age.mp/). (6111031)
13     9 not 10 (4351964)
14     11 not 12 (2001235)
15     8 not 13 (4618)
16     15 not 14 (4285)
17     limit 16 to (english language and yr="1995 -Current") (2835)
