# Supplementary material for: Immediate versus Delayed Sequential Bilateral Cataract Surgery: A Systematic Review and Meta-Analysis
Source: PLoS One. 2015 Jun 29;10(6):e0131857. doi: 10.1371/journal.pone.0131857 (PMC4485471; doi:10.1371/journal.pone.0131857)
Supplement: S3 File — (DOCX) [file pone.0131857.s003.docx]

**S3: Level 1, 2, And 3 Screening Questions**

**Level 1 Screening**

1. Does the study look at immediately bi-lateral cataract surgery?
   1. Yes
   2. No
   3. Unclear
2. Is this a research study (not an editorial, opinion, case report or a review article)?
   1. Yes
   2. No
   3. Unclear
3. Does the study look at phacoemulsification?
   1. Yes
   2. No
   3. Unclear
4. Does the study done in North America or Europe or Australia or Japan or Korea?
   1. Yes
   2. No
   3. Unclear

**Level 2 Screening**

1. Does the study look at efficacy/best corrected visual acuity/any other outcome or side-effects/complications of bi-lateral cataract surgery by phacoemulsification?
   1. Yes
   2. No
   3. Unclear
2. Is there a follow-up to adequately access efficiency or side-effects of bi-lateral cataract surgery?
   1. Yes
   2. No
   3. Unclear
3. Does the study consider the sample size of 20 or more patients?
   1. Yes
   2. No
   3. Unclear
4. Is this a research study (not a pilot study or a survey considering percentage of people suffering from blindness due to bilateral cataract surgery)?
   1. Yes
   2. No
   3. Unclear

**Level 3 Screening**

1. Are complications explicitly discussed and included in the paper?
2. Yes
3. No
4. Unclear
5. Is the study focused on bilateral cataract surgery (for example, not focused on incision, neuropsychological function, comparing unilateral lens implantation, and anesthesia)?
6. Yes
7. No
8. Unclear
9. Are complications and outcomes (ie vision, compliance, quality of life) or cost or probability of occurrence of an outcome/complication explicitly discussed and included in the paper?
10. Yes
11. No
12. Unclear
